# Developing organoids: an interview with Alice Soragni on 3D biology at the interface of treating cancers and protein aggregation

**DOI:** 10.1038/s42003-021-02390-w

**Published:** 2021-08-02

## Abstract

Alice Soragni is an Assistant Professor in the Department of Orthopedic Surgery at UCLA and a member of the Jonsson Comprehensive Cancer Center. Originally from Italy, she received her PhD in Physical Chemistry from the ETH of Zurich and a postdoc with David Eisenberg (UCLA) before starting her independent lab in December 2016. In this Q&A, Dr. Soragni tells us about her research on organoids, importance of learning from peers while starting an independent research career and of creating inclusive and diverse lab practices.

**Figure:**
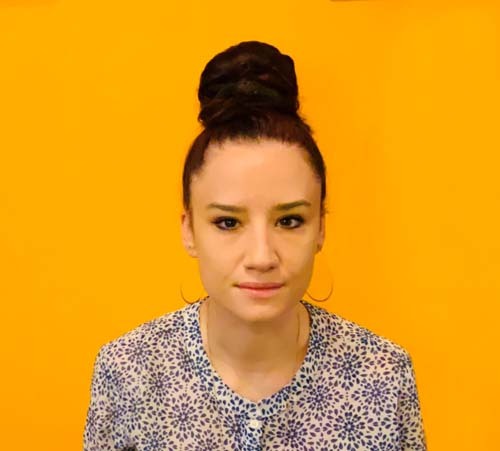
Alice Soragni

1. Can you tell us about your research interests?

I am very partial to cross-disciplinary research. My background is multidisciplinary, and I strongly believe there is strength in looking at the same problem from many different angles. My laboratory tries to solve translational challenges at the intersection of cancer biology, engineering, and structural biology. We are interested in looking at protein conformational changes in cancer cells and leveraging these for cancer therapy. On the other hand, we are also interested in developing precision medicine approaches that take advantage of the patient-derived tumor organoid models we establish. Our focus at this point is fairly exclusively on rare tumors. While some are very well studied and characterized, many rare tumors are not, to the point of not even having an established standard of care. Tumor organoids are such a handy tool that I believe will allow us to learn more about rare cancers, and help identify susceptibilities that could be exploited for therapy.

2. Your lab simultaneously focuses on two major areas of research, i.e. the effect of protein aggregation on cancer and developing organoid models for cancer biology with high-throughput screening. What do you find are the challenges and benefits of doing such cross-disciplinary research?

I actually see quite a lot of overlap, both in scope and methods. Our overarching goal is the same: we are attempting to target cancer, perhaps in less traditional ways. On the one hand, we are trying to do broad personalized organoid screenings to match existing drugs to each tumor. On the other, we are trying to increase our anti-cancer drug repertoire by developing peptides that target aberrant protein-protein interactions. While we pursue these translational objectives, we are also learning a lot of new biology about how rare cancers deal with stress and evolve under the evolutionary pressure of therapy. Really, everything we do is based on 3D biology. The work in protein aggregation is powered by 3D models as well – so there is still a lot of overlap and opportunities to learn from each other and collaborate in the lab.

3. What advice did you receive that was really important for your transition to a PI?

The best advice certainly has been to join NewPI Slack (NPIS) – a peer mentorship community, which now has over 3000 new PIs worldwide (though a majority are located in the United States). Ultimately, it’s impossible to be fully prepared for this job from the get-go. It really isn’t one but more like 10 different jobs, and you pretty much only received training in one of them. So when you start, you are thrown into the deep end and it is overwhelming but also incredibly isolating, particularly at the very beginning. There is obviously a lot to be learned from mentors who have a lot of experience, but also from peers – these are people maybe 2-3 years ahead of you at most. They have very different lived experiences yet are going through the same challenges and have timely advice that saved me from reinventing the wheel many times.

(**disclosure: I am on the board of NPIS)

4. How important is diversity to you and what do you think are the impediments for creating inclusive, equitable research labs and practices?

While there are many issues at play, one of the impediments to creating inclusive labs and research communities are the people who resist change because they benefit from a system that is convenient for some at the expense of most. Some people are conscious and intentional about it, but many others are not, and yet the outcome is just as detrimental.

There are so many incredibly talented scientists from underrepresented backgrounds that have so much going against them - their presence in science is continuously, unapologetically questioned in a system that is fundamentally biased against them. I cannot possibly understand the entirety of their experiences. But I can listen and learn. For me, it is important to contribute to implementing a system that supports underrepresented scientists and helps them achieve their goals – not only at a lab level, but at an institutional level too. Systemic changes for creating inclusive and equitable research communities can only begin when everyone has a seat at the table. I believe that there is enough space and opportunities, and nothing to be lost by giving a platform and spotlighting more voices – it is the right thing to do.

5. Organoids are both more complex than the in vitro models researchers are used to, yet not quite complex enough as in vivo models. Where do you see this field heading in the next decade?

I guess I like to say that they are not quite as complex as in vivo models… yet. The work we, and so many others, are pursuing is directed toward multi-“organ” integration. We can increase the complexity by adding more ex vivo components. People are looking at adding vasculature, kidneys, lymphoid organs, intestines, you name it. We are also trying to develop a system that includes the immune cells from the same patient we derive tumor organoids from, in order to develop an immuno-oncology screening platform.

Overall, I think we will continue to see more and more complex ex vivo integrations. It’s a challenging problem that requires different types of expertise, but an exciting one which I believe will lessen the need for in vivo experiments over the next several years.

6. Can you tell us an interesting fact about yourself that people would not commonly know?

I think most people that come across our more recent research would not necessarily anticipate that I spent a good chunk of my early career doing NMR: I definitely spent more hours than I can recall sitting in front of giant magnets (but not before a stint in microbiology). In fact, I have a PhD in Physical Chemistry (Interdisciplinary Sciences). While what I learned back then still informs my point of view, at one point I decided I was more excited about other aspects of biology, and more likely to have an impact there. And I switched from neurodegeneration to cancer research as well in that transition. I guess I have never been too afraid of change. However, 5-year-old me would be very disappointed I chose not to become a paleontologist.

*This interview was conducted by Associate Editor Anam Akhtar*.

